# miR-1322 Binding Sites in Paralogous and Orthologous Genes

**DOI:** 10.1155/2015/962637

**Published:** 2015-05-31

**Authors:** Raigul Niyazova, Olga Berillo, Shara Atambayeva, Anna Pyrkova, Aigul Alybayeva, Anatoly Ivashchenko

**Affiliations:** National Nanotechnology Laboratory, Al-Farabi Kazakh National University, 71 Al-Farabi, Almaty 050038, Kazakhstan

## Abstract

We searched for 2,563 microRNA (miRNA) binding sites in 17,494 mRNA sequences of human genes. miR-1322 has more than 2,000 binding sites in 1,058 genes with Δ*G*/Δ*G*_*m*_ ratio of 85% and more. miR-1322 has 1,889 binding sites in CDSs, 215 binding sites in 5′ UTRs, and 160 binding sites in 3′ UTRs. From two to 28 binding sites have arranged localization with the start position through three nucleotides of each following binding site. The nucleotide sequences of these sites in CDSs encode oligopeptides with the same and/or different amino acid sequences. We found that 33% of the target genes encoded transcription factors. miR-1322 has arranged binding sites in the CDSs of orthologous *MAMLD1*, *MAML2*, and *MAML3* genes. These sites encode a polyglutamine oligopeptide ranging from six to 47 amino acids in length. The properties of miR-1322 binding sites in orthologous and paralogous target genes are discussed.

## 1. Introduction

Interest in microRNAs (miRNAs) is constantly growing, and new data supplement existing knowledge about the role of these molecules in key biological processes. The main objective of these studies is to identify miRNA binding sites and evaluate their binding affinities. The characteristics of binding sites shed light on the biological role of miRNAs and have practical applications. It is possible to predict interactions between miRNAs and mRNAs and their properties by using computational methods [1]. It has been established that miRNAs bind to mRNAs predominantly in 3′-untranslated regions (3′ UTRs) [2]. They can also bind to 5′-untranslated regions (5′ UTRs) and coding domain sequences (CDSs) [3, 4]. Moreover, some miRNAs have binding sites in 5′ UTRs, CDSs, and 3′ UTRs [5]. For example, miR-3960 binding sites are mainly in CDSs, and many are positioned adjacent to each other (through one, two, three, or more nucleotides) [6]. Such mRNA fragments can consist of 2–17 binding sites. Discussed in this paper is miR-1322 which also contains multiple sites in CDSs. Clusters of miRNAs binding sites located in the CDS of genes are unexpected because proteins have specific amino acid sequences that are evolutionarily conserved. The presence of multiple binding sites in close proximity significantly increases the probability of interactions between miRNAs and mRNAs, even if mutations occur. Many miRNAs regulate the expression of genes involved in tumorigenesis [7–11]. For example, changes in miRNA concentrations are observed during the development of lung cancer [7, 8], breast cancer [9], gastrointestinal cancer [10], and other cancers [11]. The serum level of miR-1322 is a potential diagnostic biomarker for squamous cell carcinoma of the esophagus [12]. We studied the arrangement and evolution of miR-1322 binding sites in genes involved in disease.

## 2. Materials and Methods

The nucleotide sequences of precursor mRNAs (pre-mRNAs) of human genes (*Homo sapience* (Hsa)) and mammal genes (*Bos mutus* (Bmu),* Bos taurus* (Bta),* Cricetulus griseus* (Cgr),* Cavia porcellus* (Cpo),* Equus caballus* (Eca),* Felis catus* (Fca),* Gorilla gorilla* (Ggo),* Heterocephalus glaber* (Hgl),* Macaca mulatta* (Mul),* Macaca fascicularis* (Mfa),* Nomascus leucogenys* (Nle),* Pongo abelii* (Pab),* Papio anubis* (Pan),* Pan paniscus* (Ppa),* Pan troglodytes* (Ptr),* Rattus norvegicus* (Rno), and* Tupaia chinensis* (Tch)) were downloaded from NCBI GenBank (http://www.ncbi.nlm.nih.gov) in FASTA format. Nucleotide sequences of human mature miR-1322 were downloaded from the miRBase database (http://mirbase.org) [13].

Target genes for miR-1322 were determined using the MirTarget program [6], which was developed in our laboratory. This program defines the following features of binding sites: (a) the start position of an miRNA binding site with respect to the mRNA sequence; (b) the localization of miRNA binding sites in 5′ UTRs, CDSs, and 3′ UTRs of genes; (c) the free energy of hybridization (Δ*G*, kJ/mole); and (d) the schemes of nucleotide interactions between miRNAs and mRNAs. The ratio Δ*G*/Δ*G*_*m*_ (%) was estimated for each binding site, where Δ*G*_*m*_ is equal to the value of free energy of an miRNA binding with its perfect complementary nucleotide sequence. One family of miRNAs have nucleotide sequences with the level of homology of 85% or more. Therefore we used the Δ*G*/Δ*G*_*m*_ ratios of 85% or more. We also noted the positions of the binding sites on the mRNA, beginning from the first nucleotide of the 5′ UTR. The MirTarget program predicts interactions between the nucleotides of miRNAs and those of target gene mRNAs. It found bonds between adenine (A) and uracil (U), guanine (G) and cytosine (C), and G and U, as well as between A and C via a hydrogen bond [14]. The TmiROSite program was used to identify mRNA fragments that have miRNA binding sites and to define the corresponding amino acid sequences [15].

## 3. Results and Discussion

### 3.1. Features of miR-1322-3p Binding Sites

miR-1322 has a length of 19 nucleotides (nt) and a GC-content of 53%. The maximum free energy of miR-1322 binding with mRNAs is −101.9 kJ/mole. We found that miR-1322 has 2,264 binding sites on 1,058 target mRNAs with a Δ*G*/Δ*G*_*m*_ ratio of 85% or more. Of those, 160 miR-1322 binding sites are located in the 3′ UTRs of 130 genes, 215 binding sites are located in the 5′ UTRs of 109 genes, and 1,889 binding sites are located in the CDSs of 819 genes. The average number of binding sites in the CDS of a single gene is 2.3, which is almost two times higher than the average number of binding sites in 3′ UTRs.

The maximum number of sites observed in 3′ UTR is eight in* CACN1A* and five in* PDYN* and* S100A16*. The maximum number of sites in 5′ UTR was 13 in* MAB21L1*, and the* AMOT*,* BACH2*,* CAPNG*,* PIM1*,* RBM39*, and* STC1* genes have five sites. Characteristics of the clusters of five or more binding sites located in CDSs are shown in Table 1. The start points of several miR-1322 binding sites are located through three nucleotides of each other. Several such sites in mRNA form a cluster and increase the probability of binding and the ability to inhibit protein synthesis. Oligonucleotides of binding sites located in CDSs can encode polyglutamine, polyalanine, or polyserine depending on the open reading frame (Table 1). These data indicate the importance of conserved nucleotide sequences of miR-1322 binding sites and not only the amino acid sequence corresponding to oligopeptides of the encoded protein.

**Figure 1:**
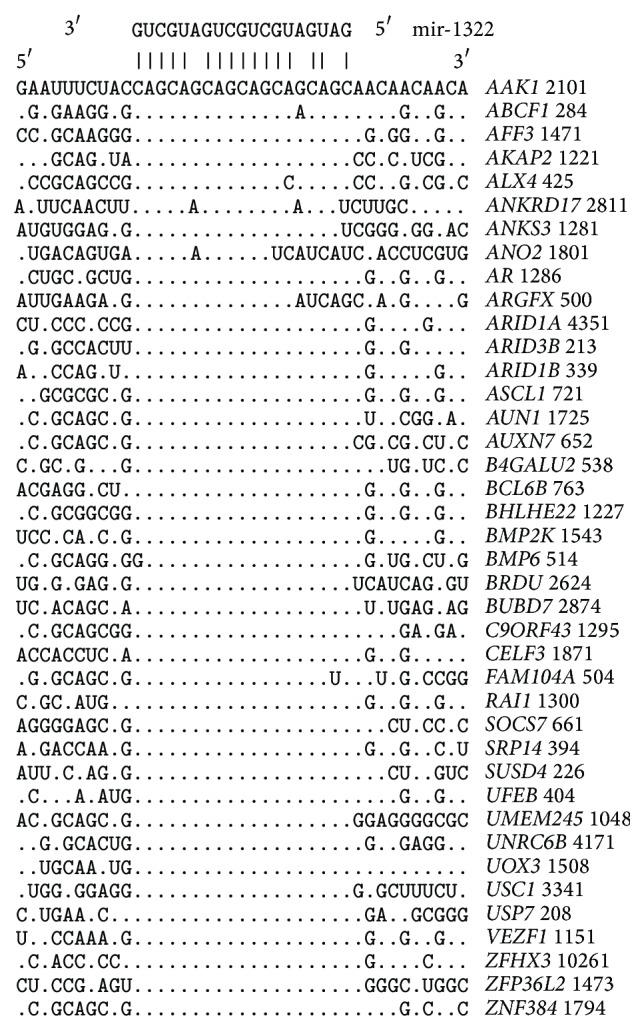
The arrangement of miR-1322 binding sites in CDSs of human target genes.

**Figure 2:**
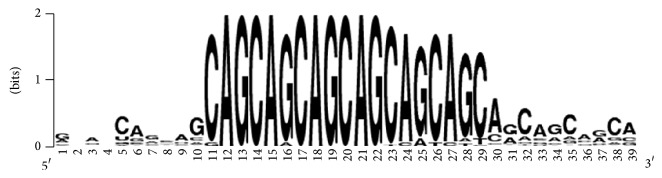
Nucleotide variation in the miR-1322 binding sites in the CDSs of human target genes.

The arranged nucleotide sequences of the CDSs contain binding sites for miR-1322 (Figure 1). The conservation of binding sites relative to the adjacent regions of CDSs is shown in Figure 2. It is important to establish the presence of miR-1322 binding sites for paralogous and orthologous mRNA sequences. Additionally, the properties of binding sites were studied for mRNA sequences of both human and other animal species.

The Δ*G*/Δ*G*_*m*_ ratio for all miR-1322 binding sites of the* ANO2* gene is 95.8%. The nucleotide fragment alignments of the CDSs containing miR-1322 binding sites for 38 genes are shown in Figure 1. Characteristics of the binding sites with start points located through three nucleotides in 5′ UTRs and 3′ UTRs are shown in Table 2. The number of binding sites in 5′ UTRs ranged from five to 13. Consequently, these untranslated regions have an increased probability of binding with miR-1322. The Δ*G*/Δ*G*_*m*_ ratio ranged from 85.4% to 91.7% (Table 2). Therefore, expression of these genes can be controlled extensively by miR-1322.

Transcription factors represent 33% of all target genes in this study (Figure 1 and Tables 1 and 2). Inhibition of the synthesis of proteins can cause diseases, including cancer. Unfortunately, experimental data on miR-1322 binding sites are insufficient; however, some previous studies confirm the high efficacy of the predictions of the MirTarget program developed in our laboratory. For example, downregulation of* ECRG2* and* TCA3* is associated with squamous cell carcinoma of the esophagus (ESCC) via miR-1322 [12]. ECRG2 can act as a tumor suppressor, regulating protease cascades during carcinogenesis and the migration and invasion of esophageal cancer cells [16].

### 3.2. Binding Sites in Paralogous and Orthologous mRNAs of the MAML Gene Family

The relationship between paralogous and orthologous mRNAs of the* MAML* gene family was considered an example of adaptation of gene expression to the action of miR-1322.* MAMLD1* encodes a mastermind-like domain-containing protein, which can act as a transcriptional coactivator [17]. Both* MAML2* and* MAML3* stabilize the DNA-binding complex RBP-J/CBF-1 and the Notch intracellular domains that are signaling intermediates [18]. Higher* MAML2* expression is observed in several B cell-derived lymphoma types, including classical Hodgkin's lymphoma cells, more than in normal B cells [19].

**Figure 3:**
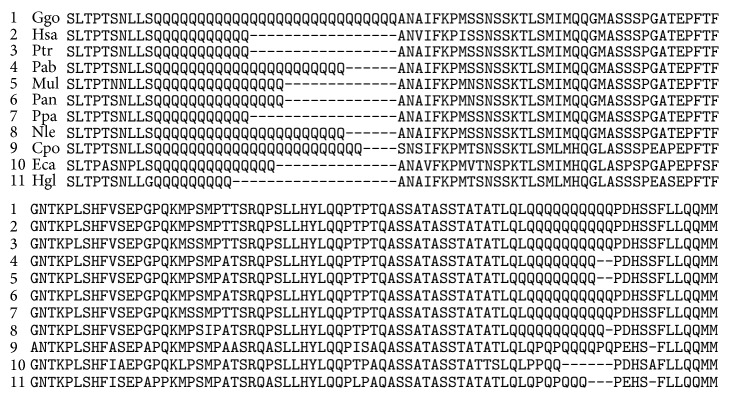
Conserved amino acid sequences containing polyglutamine in orthologous* MAMLD1*.

Various paralogous genes are targets for miR-1322. Two regions contain multiple miR-1322 binding sites in* MAMLD1* (Figure 3). The first region consists of eight sites and the second region consists of four sites. They were in domains (oligopeptides) consisting of 11 and 10 glutamine residues in the corresponding proteins, respectively.

The number of amino acids in orthologous proteins depends on the species (Figure 3). For example, for the first region, there are 28 glutamine residues in Ggo and nine residues in Hgl. Ten glutamine residues of Hsa, Ggo, and Ptr mRNAs to six of Eca mRNA were identified in the second region. In this case, the binding site of horse mRNA encoded proline in the associated protein.

**Figure 4:**
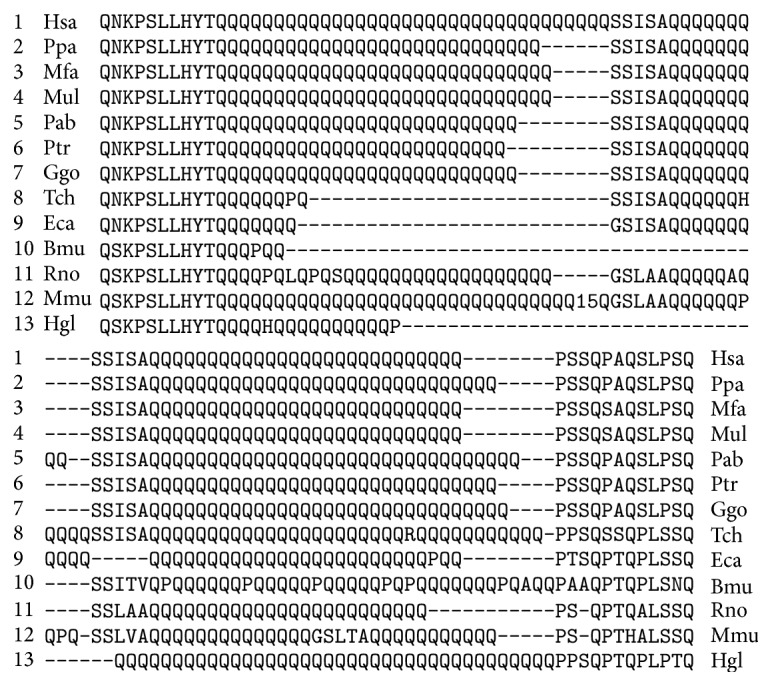
Conserved amino acid sequences containing polyglutamine in orthologous* MAML2*. Note: the number “15” indicates the number of glutamine residues in a site position of Mmu protein.

**Figure 5:**
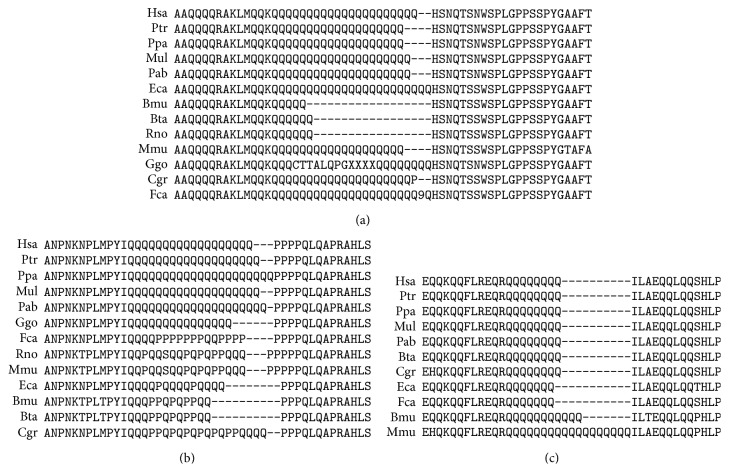
Conserved amino acid sequences containing polyglutamine in orthologous* MAML3*. Note: the number “9” indicates the number of glutamine residues in a site position of Fca protein (a).

miR-1322 binding sites in orthologous* MAML* mRNAs are highly conserved. Orthologous* MAML* proteins have conserved amino acid sequences containing polyglutamine (Figures 3–5). Orthologous miRNAs are not identified in most animals except* Pan troglodytes* (chimpanzee) and* Pongo pygmaeus* (orangutan); however, some other miRNAs are identical or very similar to the corresponding human miRNAs. Therefore, human miRNAs were used for the subsequent identification of conserved binding sites. Oligonucleotides containing CAG repeats represent the miR-1322 binding site of the mRNA that encoded a long polyglutamine sequence in the corresponding protein. Oligonucleotides encoding polyglutamine are located in the conserved protein domain.

The CDS of the human* MAML2* gene also has two regions with miR-1322 binding sites and encodes oligopeptides containing 47 and 27 glutamine residues (Figure 4). The number of glutamine residues in the oligopeptides is varied depending on the species. For example, there are six glutamine residues in the first oligopeptide region of the cow protein and 24 residues in the second region of the rat protein.

The CDS of the human* MAML3* gene has three regions that contain miR-1322 binding sites, and it encodes oligopeptides containing 21, 18, and eight glutamine residues. Some amino acids were lacking in the domains of* MAML3*, depending on the species (Figures 5(a)–5(c)).

The presence of multiple miR-1322 binding sites in* MAMLD1*,* MAML2*, and* MAML3* demonstrates their interactions. The expression of these genes has become increasingly important because the studied organisms were separated by tens of millions of years. The presence of multiple regions containing miR-1322 binding sites in* MAMLD1*,* MAML2*, and* MAML3* genes shows a strong dependence of their expression via miR-1322.

The glutamine-containing regions play an important role in the development of different diseases, according to previous literature. It is possible that changes in the dependence of the interactions between miR-1322 and* MAMLD1*,* MAML2*, and* MAML3* are interconnected.

## Figures and Tables

**Table 1:** Characteristics of miR-1322 binding sites located through three nucleotides in the CDSs of some mRNAs. The number of binding sites in the mRNA fragment is indicated within parentheses.

Gene	Position of binding sites, nt	Δ*G*/Δ*G*_*m*_, %	Oligopeptide
*AFF3 *	1471–1486 (6)	85.4 ÷ 87.5	SSSSSSSGSSS
*AR *	1286–1334 (17)	87.5	QQQQQQQQQQQQQQQQQQQQQQ
*ARID3B *	213–225 (5)	87.5	QQQQQQQQQQ
*ASCL1 *	724–742 (8)	87.5	QQQQQQQQQQQA
*ATN1 *	1692–1731 (13)	87.5 ÷ 91.7	QQQQQQQQQQQQQQQQQH
*ATXN1 *	1559–1592 (12)	85.4 ÷ 91.7	QQQQQQQQQQQQHQHQQ
	1604–1631 (10)	87.5 ÷ 89.6	QQQQQQQQQQQQQQH
*ATXN2 *	657–684 (10)	87.5	QQQQQQQQQQQQQQQ
	699–714 (6)	87.5 ÷ 89.6	QQQQQQQQQPР
*ATXN7 *	637–658 (8)	85.4 ÷ 89.6	QQQQQPPPPQPQ
*BCL6B *	763–775 (5)	85.4 ÷ 87.5	SSSSSSSSSS
*BHLHE22 *	1224–1236 (5)	85.4 ÷ 87.5	GSSSSSSSSS
*BMP2K *	1543–1555 (5)	87.5	QQQQQQQQQQ
	1600–1615 (6)	85.4 ÷ 91.7	QQQQQQQQHHH
*C9orf43 *	1283–1298 (6)	85.4 ÷ 87.5	QQQRQQQQQQQ
*CELF3 *	1871–1883 (5)	87.5	QQQQQQQQQQ
*E2F4 *	980–1007 (10)	85.4 ÷ 87.5	SSSSSSSSSSSSSNS
*EP400 *	8333–8363 (11)	87.5	QQQQQQQQQQQQQQQQ
*FAM155A *	732–753 (8)	85.4 ÷ 87.5	QQQQRQQQQQQQ
*FAM157A *	408–432 (9)	87.5	QQQQQQQQQQQQQQ
*FAM157B *	414–435 (8)	85.4 ÷ 87.5	RQQQQQQQQQQQ
*HTT *	196–247 (19)	85.4 ÷ 89.6	QQQQQQQQQQQQQQQQQQQQQQQ
*IRF2BPL *	1249–1267 (7)	87.5	QQQQQQQQQQQQ
*IRS1 *	2088–2100 (5)	85.4 ÷ 87.5	SSSSSSSNAV
*KCNN3 *	512–539 (10)	87.5 ÷ 91.7	QQQQQQQQQQQQQQP
*KIAA2018 *	4794–4815 (8)	87.5	QQQQQQQQQQQQQ
*MAGI1 *	1759–1771 (5)	87.5	QQQQQQQQQQ
*MAML2 *	3064–3091 (10)	87.5	QQQQQQQQQQQQQQQ
*MAML3 *	2219–2264 (16)	87.5	QQQQQQQQQQQQQQQQQQHSN
	2678–2690 (5)	87.5 ÷ 91.7	QQQQQPPPPQ
*MED15 *	710–722 (5)	87.5 ÷ 91.7	QQQQQQQQHL
	830–848 (7)	87.5	QQQQQQQQQQQQ
*MEF2A *	1836–1860 (9)	85.4 ÷ 89.6	GFQQQQQQQQQQQP
*MLLT3 *	729–741 (5)	85.4 ÷ 87.5	SSSSSSSSSS
	762–774 (5)	85.4 ÷ 87.5	SSSSSSSSSS
*MN1 *	2524–2539 (6)	87.5	QQQQQQQQQQQ
*MPRIP *	622–643 (8)	87.5 ÷ 91.7	SSSSSSSSSSSIP
*NAP1L3 *	549–561 (5)	85.4 ÷ 87.5	GSGSSSSSSG
*NCOA3 *	4023–4038 (6)	87.5	QQQQQQQQQQQ
*NCOA6 *	1126–1138 (5)	87.5	QQQQQQQQQQ
*NCOR2 *	1812–1830 (7)	87.5 ÷ 91.7	QQQQQQQQQQQQ
*POLG *	408–429 (8)	85.4 ÷ 87.5	QQQQQQQQQQQQQ
*POU6F2 *	701–719 (7)	85.4 ÷ 89.6	QQQQQQQQQQPP
*PRPF40A *	785–797 (5)	85.4	AAAAAAAAAA
*RAI1 *	1300–1324 (9)	87.5	QQQQQQQQQQQQQQ
*SALL1 *	590–602 (5)	85.4 ÷ 87.5	SSSSSSSSSG
*SCAF4 *	3303–3315 (5)	87.5 ÷ 91.7	QQQQQQQPPP
*SMARCA2 *	765–795 (11)	87.5	QQQQQQQQQQQQQQQQ
*SRP14 *	394–406 (5)	87.5 ÷ 91.7	AAAAAAAAAP
*TBP *	468–480 (5)	87.5	QQQQQQQQQQ
	501–546 (16)	87.5	QQQQQQQQQQQQQQQQQQQQQ
*THAP11 *	611–629 (7)	87.5 ÷ 91.7	QQQQQQQQQQQQ
*TNS1 *	2348–2363 (6)	87.5 ÷ 91.7	QQQQQQQQQPR
*VEZF1 *	1151–1175 (9)	87.5	TSNQKQQQQQQQQQ
*ZNF384 *	1770–1806 (13)	87.5 ÷ 91.7	QQQQQQQQQQQQQQQQPP

**Table 2:** The arrangement of miR-1322 binding sites in 5′ UTRs and 3′ UTRs human target genes.

Gene	Position of binding sites, nt	Δ*G*/Δ*G*_*m*_, %
*BACH2 *	25–43 (7)	85.4 ÷ 87.5
*CACNA1A* ^*^	7170–7191 (8)	87.5
*CAPN6 *	118–136 (7)	85.4 ÷ 87.5
*CNKSR2 *	178–199 (8)	87.5 ÷ 89.6
*GLS *	53–86 (12)	85.4 ÷ 89.6
*MAB21L1 *	342–378 (13)	87.5
*PDYN* ^*^	1413–1425 (5)	85.4 ÷ 87.5
*PIM1 *	103–118 (7)	85.4 ÷ 89.6
*RBM39 *	323–335 (5)	87.5 ÷ 91.7

Note: the symbol “^*^” indicates binding site localization in the 3′ UTR.
